# GRAF-pop: A Fast Distance-Based Method To Infer Subject Ancestry from Multiple Genotype Datasets Without Principal Components Analysis

**DOI:** 10.1534/g3.118.200925

**Published:** 2019-05-31

**Authors:** Yumi Jin, Alejandro A. Schaffer, Michael Feolo, J. Bradley Holmes, Brandi L. Kattman

**Affiliations:** * National Center for Biotechnology Information, National Institutes of Health, Department of Health and Human Services, Bethesda, Maryland 20894 and; † Cancer Data Science Laboratory, National Cancer Institute, National Institutes of Health; Department of Health and Human Services; Bethesda, Maryland 20892

**Keywords:** ancestry inference, population structure, admixture mapping, GWAS, barycentric coordinates

## Abstract

Inferring subject ancestry using genetic data is an important step in genetic association studies, required for dealing with population stratification. It has become more challenging to infer subject ancestry quickly and accurately since large amounts of genotype data, collected from millions of subjects by thousands of studies using different methods, are accessible to researchers from repositories such as the database of Genotypes and Phenotypes (dbGaP) at the National Center for Biotechnology Information (NCBI). Study-reported populations submitted to dbGaP are often not harmonized across studies or may be missing. Widely-used methods for ancestry prediction assume that most markers are genotyped in all subjects, but this assumption is unrealistic if one wants to combine studies that used different genotyping platforms. To provide ancestry inference and visualization across studies, we developed a new method, GRAF-pop, of ancestry prediction that is robust to missing genotypes and allows researchers to visualize predicted population structure in color and in three dimensions. When genotypes are dense, GRAF-pop is comparable in quality and running time to existing ancestry inference methods EIGENSTRAT, FastPCA, and FlashPCA2, all of which rely on principal components analysis (PCA). When genotypes are not dense, GRAF-pop gives much better ancestry predictions than the PCA-based methods. GRAF-pop employs basic geometric and probabilistic methods; the visualized ancestry predictions have a natural geometric interpretation, which is lacking in PCA-based methods. Since February 2018, GRAF-pop has been successfully incorporated into the dbGaP quality control process to identify inconsistencies between study-reported and computationally predicted populations and to provide harmonized population values in all new dbGaP submissions amenable to population prediction, based on marker genotypes. Plots, produced by GRAF-pop, of summary population predictions are available on dbGaP study pages, and the software, is available at https://www.ncbi.nlm.nih.gov/projects/gap/cgi-bin/Software.cgi.

Inference of individual ancestry from genetic data arises in genome wide association studies (GWAS), personalized medicine, and human migration studies. Several reviews on ancestry inference methods are available (Lawson and Falush 2012; Liu *et al.* 2013; Padhukasahasram 2014; Wollstein and Lao 2015; Novembre and Peter 2016; Hellwege *et al.* 2017).

The “(global) problem of ancestry inference” of human genetics takes as input genotype data on *M* markers for *N* subjects and produces as output some sort of clustering or assignment of the *N* subjects into populations. There have been two popular classes of solutions to this problem: model-based and distance-based methods.

Model-based approaches, represented by the software packages STRUCTURE (Pritchard *et al.* 2000), fastSTRUCTURE (Raj *et al.* 2014), ADMIXTURE (Alexander *et al.* 2009), and FRAPPE (Tang *et al.* 2006), estimate ancestral proportions based on a statistical model, *e.g.*, individuals being randomly drawn from *K* distinct populations and may also use the technique of Hidden Markov Models (HMMs). Distance-based methods are based on embedding points in a high-dimensional space and combining algebraic and geometric tools to represent key features. Distance-based methods use principal components analysis (PCA) (Patterson *et al.* 2006; Price *et al.* 2006), multidimensional scaling (MDS) (Purcell *et al.* 2007), spectral graph theory (Lee *et al.* 2010), or network theory (Greenbaum *et al.* 2016). The focus of our study is a new distance-based method, which we have implemented in freely available software, called GRAF-pop.

PCA-based approaches are the most used model-free ancestry inference methods. PCA analyzes a pairwise genetic similarity or genetic distance matrix between individuals and finds ancestral clusters by projecting the data to a lower-dimensional space. Compared to model-based methods, PCA-based approaches are usually faster and easier to use. Model-based methods estimate parameters using computationally intensive algorithms such as Markov Chain Monte Carlo algorithm (Pritchard *et al.* 2000) or maximum likelihood estimation (Tang *et al.* 2005; Tang *et al.* 2006; Alexander *et al.* 2009). Multiple parameters need to be tuned to run model-based software. Choosing the number of populations is difficult (Alexander *et al.* 2009), and usually also requires knowledge of the population’s history.

The first PCA methods such as EIGENSTRAT, have running times that grow as the square of *N* (more formally O(*MN^2^*)). Advances in random matrix theory led to the implementation of asymptotically faster methods with running time linearly proportional to *N*, such as FlashPCA (Abraham and Inouye 2014; Abraham *et al.* 2017) and FastPCA (Galinsky *et al.* 2016). PCA analysis *across* datasets is not necessarily robust.

PC coordinates in the PCA plots are not directly interpretable (Lee *et al.* 2010) in contrast to the coefficients estimated by model-based methods. In addition, unlike model-based methods, traditional distance-based approaches do not necessarily assign individuals to populations or estimate ancestral proportions for admixed individuals.

Model-based and PCA-based methods can work well for datasets with samples genotyped for the same set of variants using the same genotyping or sequencing platform. However, researchers often wish to do meta-analyses combining multiple datasets genotyped using different methods. With the establishment of public genotype databases, such as the database of Genotypes and Phenotypes (dbGaP) (Mailman *et al.* 2007) and the European Genotype-phenotype Archive (EGA) (Lappalainen *et al.* 2015), investigators now can access genotype datasets of millions of individuals. Most PCA-based methods, as well as model-based approaches, generate different ancestry results for the same individual when different individuals are included in the datasets being analyzed. Moreover, PC scores are not comparable when different sets of markers are analyzed.

Because of the public availability of outside data that can guide ancestry inference, several research groups considered modifications to problem of ancestry inference, which we describe here together as methods that incorporate reference data. The reference data for these methods typically consist of genotypes of individuals whose ancestries are extrememly reliable and not very admixed (*e.g.*, the original HapMap samples). One way to use such reference data is to find ancestry informative markers (AIMs) using the subjects with reliable ancestries and to use AIMs to infer ancestry of other subjects. Many AIM panels have been built, especially for forensic analyses (Halder *et al.* 2008; Nassir *et al.* 2009; Galanter *et al.* 2012; Daya *et al.* 2013; Kidd *et al.* 2014; Bulbul *et al.* 2018; Wang *et al.* 2018; Zhao *et al.* 2019). Another way to infer ancestry with reference data can be seen in ADMIXTURE and FRAPPE that perform supervised analyses using reference panels to improve the estimation accuracy (Tang *et al.* 2006; Alexander *et al.* 2009; Thornton *et al.* 2014; Conomos *et al.* 2015).

A third formulation of methods using reference data, which we consider here, is to infer ancestry with the aid of only *the summary data* of the reference panels. This formulation is attractive because one does not need to spend effort or obtain permission to download individual genotypes. iAdmix (Bansal and Libiger 2015) is a model-based method that infers ancestry using a reference set of population allele frequencies. ADMIXTURE has been upgraded to estimate individual ancestry by projecting the new samples to the pre-calculated population structure of reference panels (Shringarpure *et al.* 2016). Similarly, for PCA-based methods, subject ancestry can be inferred by projecting samples in one dataset to the SNP weights calculated using another dataset. PCAiR (Conomos *et al.* 2015) partitions subjects in the target dataset into a “reference” subset containing individuals known to be unrelated and another subset containing all other individuals, and calculates SNP weights using the reference subset and applies the weights to all individuals. SNPweights (Chen *et al.* 2013) and FastPop (Li *et al.* 2016) use fixed sets of pre-selected SNPs from outer reference populations to calculate SNP weights and infer subject ancestry for target samples by projecting their genotypes to these SNP weights. Using SNP weights pre-calculated from the same reference populations makes the results comparable across target datasets and allows the software to execute faster.

The previous methods using reference data employ a fixed set of SNPs from reference panels. These methods require that the target samples have genotypes available for most of these SNPs. This requirement is hard to meet when analyzing multiple datasets downloaded from databases like dbGaP. To date, dbGaP has genotypes of more than a million subjects spanning hundreds of studies, genotyped using many different platforms. No SNP is included in all genotyping platforms found in dbGaP. Nevertheless, both dbGaP staff (for harmonization and QA/QC) and outside researchers (so they can reanalyze the data), want to infer ancestry of dbGaP subjects across studies.

No previous methods for ancestry inference are robust in datasets with high rates of non-randomly missing genotypes. Most existing tools filter out individuals with high genotype missing rates, *e.g.*, 5% or more. When encountering missing genotypes in calculations, existing methods, *e.g.*, EIGENSTRAT (Price *et al.* 2006), SNPweights (Chen *et al.* 2013), and AIPS (Byun *et al.* 2017), usually replace them with the mean genotype values. As more missing genotypes are replaced, the more likely that an individual is predicted to have an average ancestry background, where average depends on the other members of the target or reference dataset used.

To find duplicate samples and closely related subjects across studies in dbGaP, we previously developed a statistical method and software tool called GRAF (standing for Genetic Relationship And Fingerprinting) (Jin *et al.* 2017). One contribution of GRAF was to define a set of 10,000 unlinked, autosomal biallelic SNPs with high minor allele frequency (MAF), good coverage on many of the widely used genotyping platforms, and other desirable characteristics. GRAF extracts genotypes of these “fingerprint SNPs” for all samples.

Here, we describe GRAF-pop, a new distance-based method for ancestry inference using reference, that uses the fingerprint genotypes extracted by GRAF and 1) does not assume that the reference populations and target samples have been genotyped at mostly the same markers, 2) does not impute missing genotypes, 3) only requires allele frequencies of reference populations, and 4) does not use PCA. GRAF-pop can give reliable population assignments when the rate of missing genotypes far exceeds 5%. GRAF-pop makes accurate ancestry estimations even if the missing genotypes are non-random. The main continental populations can be well separated from one another even when there are only 100-200 fingerprint SNPs with genotypes. GRAF-pop is fast, with running time linear in the product of number of individuals and SNPs. In addition, GRAF-pop incorporates a visualization tool to plot all individuals into the same plot and uses the same standards to assign them into different populations, regardless of the genotyping platform.

## Methods

### Use of dbGaP data for the development of validation tools

GRAF and GRAF-pop have been developed to improve quality control in curation, and to harmonize population values across studies. NCBI staff have access to data within dbGaP for the purposes of managing data, QC, and tool development, such as the development of GRAF previously, and here GRAF-pop. The analyses shown here involve six dbGaP studies: phs000050 (HapMap), phs000403, phs000420, phs000456, phs000517, phs000788. The study-reported population summary information for these studies are published both in literature, and on public dbGaP. This manuscript was reviewed in accordance with NIH Genomic Data Sharing Policy governace.

### Reference population data and subject group data from within dbGaP

Herein, the phrase “study-reported population” refers to the population term assigned to a subject submitted to dbGaP by the group producing/collecting data. The study-reported population terms and granularity of the terms vary across studies and may have been either collected from participant interviews or questionnaires or inferred computationally using genotypes. Some studies submitted to dbGaP do not submit any population values.

The phrase “reference populations” refers to five large sets of dbGaP subjects with the following study-reported population values: (1) White, Caucasian, European, European American, and other equivalent terms (2) Black, African, African American, Ghana, Yoruba, etc. (3) Asian, East Asian, Chinese, Japanese, etc. (4) Asian Indian, Pakistani; (5) Mexican, Latino. After closely related subjects and outliers were removed using GRAF, allele frequencies were calculated for each reference population.

The term “subject group(s)” refers to smaller sets of subjects from dbGaP: 8,475 study-reported CEU (denoted E), 904 individuals from Ghana (denoted F), 7,612 Chinese, and 4,138 Japanese (combined and denoted A). The subject groups are subsets of reference populations 1, 2, and 3, respectively, in which the study-reported population is especially trustworthy.

### Clustering subjects using genetic distances

Suppose there are *K* mutually exclusive, random-mating reference populations, with *n_j_* individuals for each population *j*, and $\overset{K}{\underset{j=1}{\sum }}n_{j}=N$. Consider *S* independent, biallelic SNPs. For each SNP *l* and population *j*, suppose the reference and alternate allele frequencies are *p_jl_* and *q_jl_*, respectively. The fingerprint SNPs used by GRAF and GRAF-pop were previously selected to be spread out, so that no pairs have substantial linkage disequilibrium and on the initial test sets were in Hardy-Weinberg equilibrium. Let *g_il_* ∈{*0*, *1*, *2*} be the count of reference alleles of subject *i* at SNP *l*. The probabilities to have each *g_il_* value are:

$$P\left(g_{il}\text{|}population=j\right)=\{\begin{array}{c} q_{il}^{2}\;\;\;\;\;if\;g_{il}=0\\ 2p_{il}q_{il}\;\;if\;g_{il}=1\\ p_{il}^{2}\;\;\;\;\;if\;g_{il}=2 \end{array}$$

Denoting *g_i_* as the genotype of the subject *i* over all the *S* SNPs, we have

$$P\left(g_{i}\text{|}population=j\right)=\overset{S}{\underset{l=1}{\prod }}P\left(g_{il}\text{|}population=j\right)$$

In principle, the probability that subject *i* belongs to population *j* could be calculated using Bayes’ theorem:

$$P\left(population=j\text{|}g_{i}\right)=\frac{n_{j}P\left(g_{i}\text{|}population=j\right)}{\sum_{k=1}^{K}n_{k}P\left(g_{k}\text{|}population=k\right)}\;$$ (1)

In practice, most individuals cannot be unambiguously classified into populations due to admixture. Equation (1) does imply that the probability *P(g_i_|population = j)* reflects the similarity between subject *i* and population *j*. Taking negative logarithm and normalizing it on number of SNPs with genotypes, we define a “distance”:

$$D_{ij}=-\frac{1}{S}\;ln\left(\overset{S}{\underset{l=1}{\prod }}P\left(g_{il}\text{|}population=j\right)\right)$$

It can be rewritten as:

$$D_{ij}=-\frac{1}{S}\;\overset{S}{\underset{l=1}{\sum }}\left[g_{il}\;\ln\left(p_{jl}\right)+\left(2-g_{il}\right)\ln\left(q_{jl}\right)+g_{il}\left(2-g_{il}\right)\ln\left(2\right)\right]\;$$ (2)

Since *D_ij_* is greater when the genotypes of subject *i* are more different from the genotypes of population *j*, we use *D_ij_* as an estimate of the genetic distance between subject *i* and population *j*.

For each subject *i*, we can calculate *K D_ij_* values, and represent them as a point in *K*-dimensional space. If we plot all subjects in the *K*-dimensional space, subjects will be clustered based on their genetic similarities. We treat the centroids of the *K* reference population clusters as vertices of a (*K-1*)-dimensional simplex (*e.g.*, a triangle if *K = 3*).

### Estimating ancestry proportions using barycentric coordinates

Assuming all subjects are admixtures of *R* (1 < *R* ≤ *K*) ancestry populations, we can define a simplex based on the *R* centroids and then use *barycentric coordinates* (Ungar 2010) (defined below) to estimate ancestry proportions by mapping each subject to a point relative to the (R-1)-dimensional simplex. We select *R* groups of subjects with genotypes of the same set of *S* SNPs mentioned above. Suppose all subjects in each group have one of the *R* ancestries. For each subject, we calculate the *D* values with reference to *R-1* populations and represent that subject by a position in (*R*-1)-dimensional space. For each ancestry group, we calculate the centroid of all its subjects.

The *R* centroids, denoted ***V_1_***, …, ***V****_R_*, become vertices of a simplex. For any point ***Q*** representing a subject, we calculate the unique absolute barycentric coordinates (*λ_1_*, …, *λ_R_*) for ***Q*** by solving the following equations:

$$Q=\lambda_{1}V_{1}+...+\;\lambda_{R}V_{R}\;$$

$$\overset{R}{\underset{r=1}{\sum }}\lambda_{r}=1$$

The unique solution of the equations can be found by taking determinants of some 3×3 matrices when *R* = 3, as described below, or by an equivalent geometric method (Ungar 2010). We use R = 3, rather than R = 5 partly because 3 dimensions are the most that are easy to visualize. If ***Q*** is within the simplex, then barycentric coordinates are all positive. Letting *P_r_* be the proportion of ancestry *r*, we estimate the proportion as *P_r_* = *λ_r_*. In general ***Q*** may fall outside of the simplex, so we estimate *P_r_* as follows:

1. Let *λ’_r_* = *max*(0, *λ_r_*), ∀r∈{*1*, .., *R*}
2. Set *P_r_* as:
  $$P_{r}=\frac{\lambda_{r}^{’}}{\sum_{r=1}^{R}\lambda_{r}^{’}}$$

Starting from a triangle, where each vertex represents one of the three HapMap populations, SNPweights (Chen *et al.* 2013) uses the same algebra to compute ancestry proportions for points inside the triangle, but handles points outside the triangle differently.

### Normalizing scores using barycentric coordinates

In previous subsections, we assumed that all subjects have genotypes on the same SNPs. In dbGaP usage, genotypes are missing because different genotyping platforms were used in different dbGaP studies. We normalize the *D* values when there are missing genotypes. For an ancestry group *r*, suppose the two allele frequencies of SNP *l* are *u_rl_* and *v_rl_*. If we compare all subjects in *r* to population *j* over *S* SNPs, the mean genetic distance is expected to be:

$$E_{D\_all}=E\left(D_{rj}\right)=-\frac{1}{S}\;\overset{S}{\underset{l=1}{\sum }}\left[u_{rl}^{2}\;\ln\left(p_{jl}^{2}\right)+2u_{rl}v_{rl}\text{ln}\left(2p_{jl}q_{jl}\right)+v_{rl}^{2}\;\ln\left(q_{jl}^{2}\right)\right]$$ (3)

The subscript “all” conveys that all 10,000 SNPs are used at this stage. For a subject *i*, if all *S* SNPs have genotypes, we can 1) use Equation (3) to calculate the expected centroids for the *R* ancestry groups, 2) use the simplex formed by these centroids, and 3) calculate the ancestry proportions of each new subject as barycentric coordinates with respect to the simplex. If there are missing genotypes, we ignore the SNPs with genotypes missing, and calculate the genetic distance from subject *i* to each population *j*.

Suppose subject *i* has genotypes for *S*’ (*S’ < S)* fingerprint SNPs, *i.e.*, *l_1_*, *l_2_*, *…*, *l_S_*_’_. We use an equation like Equation (3) to calculate the expected distance *E_Di_*, from each group *r* to population *j*, for the subset of SNPs genotyped for subject *i*.

$$E_{Di}=-\frac{1}{S^{\prime }}\;\overset{S^{\prime }}{\underset{m=1}{\sum }}\left[u_{rl_{m}}^{2}\ln\left(p_{jl_{m}}^{2}\right)+2u_{rl_{m}}v_{rl_{m}}\text{ln}\left(2p_{jl_{m}}q_{jl_{m}}\right)+v_{rl_{m}}^{2}\ln\left(q_{jl_{m}}^{2}\right)\right]$$ (4)

Using *E_Di_* values of the *R* ancestry groups as vertices, we can build a simplex and use it as a reference to calculate the barycentric coordinates for subject *i*. The barycentric coordinates are converted back to the Cartesian coordinates using *E_D_all_* values as references for plotting.

### Implementing the ancestry inference method Into the GRAF software package

The above method is implemented in the GRAF-pop feature of the GRAF software package (version 2.3). As in GRAF 1.0, the main program is implemented in C++, auxiliary programs are implemented in Perl, and graphic displays rely on the Perl GD Graphics Library (http://search.cpan.org/∼lds/GD-1.38/GD.pm).

We use the same 10,000 well-separated, autosomal, biallelic SNPs used in GRAF v1.0 (Jin *et al.* 2017) to determine subject ancestry. Five reference populations, listed above, are used to calculate genetic distances. The allele frequencies at these SNPs (*p_jl_* and *q_jl_*) are estimated using the subjects submitted to dbGaP and reported by the submitters as having these ancestry backgrounds. For each subject *i*, we calculate five *D* values: *D_i1_*, *D_i2_*, *D_i3_*, *D_i4_*, *D_i5_*, for the five reference populations.

The first three *D* values, *D_i1_*, *D_i2_* and *D_i3_*, are treated as the *x*, *y*, *z* Cartesian coordinates for each subject *i*, and are used for calculating barycentric coordinates. Three subject groups (E, F, A) are used to build the reference triangle, denoted as ΔEFA, whose vertices are the centroids of these three groups. This triangle is mapped to the x-y plane by translation and rotation, so that the side FA is parallel to the *x*-axis. The transformed triangle is used for calculating the barycentric coordinates and ancestry proportions. All 3-D points representing subjects being checked are also projected onto the plane where ΔEFA is located. We use numerical subscripts 1,2,3 in equations and subscripts E, F, A, when referring to the triangle.

We estimate the ancestry proportions for ancestries {E, F, A}, for each subject using barycentric coordinates. Let (*x_i_*, *y_i_*) be the Cartesian coordinates of subject *i* on the 2-D plane after transformation, and (*x_e_*, *y_e_*), (*x_f_*, *y_f_*), (*x_a_*, *y_a_*) be the coordinates of the three vertices of ΔEFA. Denote matrix ***T*** as:

$$T=\;\left(\begin{array}{c} 1\;x_{e}\;y_{e}\\ 1\;x_{f}\;y_{f}\\ 1\;x_{a}\;y_{a} \end{array}\right)$$

The barycentric coordinates (*λ_ie_*, *λ_if_*_,_, *λ_ia_*) of subject *i* with respect to ΔEFA are calculated using the following equations:

$$\lambda_{ie}=\frac{\left|\begin{array}{c} 1\;x_{i}\;y_{i}\\ 1\;x_{f}\;y_{f}\\ 1\;x_{a}\;y_{a} \end{array}\right|}{\text{det}(T)};\;\lambda_{if}=\frac{\left|\begin{array}{c} 1\;x_{e}\;y_{e}\\ 1\;x_{i}\;y_{i}\\ 1\;x_{a}\;y_{a} \end{array}\right|}{\text{det}(T)};\;\lambda_{ia}=\frac{\left|\begin{array}{c} 1\;x_{e}\;y_{e}\\ 1\;x_{f}\;y_{f}\\ 1\;x_{i}\;y_{i} \end{array}\right|}{\text{det}(T)}$$ (5)

The ancestry proportions *P_ie_*, *P_if_*, *P_ia_* are calculated using the following equation:

$$P_{im}=\frac{\text{max}(0,\;\lambda_{im})}{\text{max}(0,\;\lambda_{ie})+\text{max}(0,\;\lambda_{if})+\text{max}(0,\;\lambda_{ia})}\;$$ (6)

where *P_im_* ∈{*P_ie_*, *P_if_*, *P_ia_*} and *λ_im_* ∈{*λ_ie_*, *λ_if_*, *λ_ia_*}.

In GRAF-pop, we use the expected *D* values calculated using Equation (3) to find the vertices of ΔEFA, and use it as the reference triangle to calculate barycentric coordinates. When there are missing genotypes, we first calculate the barycentric coordinates using the SNPs with genotypes, then map the coordinates onto the reference triangle when no genotypes are missing. Specifically, we do the following steps to normalize the genetic distances and plot the results:

1. For each subject group *U* ∈*{E*, *F*, *A}*, calculate the expected genetic distances *D_U1_*, *D_U2_*, *D_U3_*, to the first three reference populations, using Equation (3) for the full set of 10,000 fingerprint SNPs.
2. Represent each group with a 3-D point in space by treating *D_U1_*, *D_U2_*, *D_U3_* as the *x*, *y*, *z* Cartesian coordinates. Build a triangle by connecting the three points representing the three subject groups.
3. Rotate and translate the triangle so that it is in the plane *z* = 0, and side FA is parallel to the *x*-axis. Denote this triangle as ΔEFA_0_, to be used as the reference triangle for calculating barycentric coordinates.
4. Given a subject *i*, find the subset, *T*, of fingerprint SNPs with genotypes for this subject.
5. For each subject group *U* ∈*{E*, *F*, *A}*, calculate the expected genetic distances *D_U1T_*, *D_U2T_*, *D_U3T_*, to the first three reference populations using Equation (4) for subset *T*. Represent each group with a 3-D point in the space.
6. Calculate the genetic distances *D_i1_*, *D_i2_* and *D_i3_* from subject *i* to the first three reference populations. Represent subject *i* as a 3-D point, ***Q*,** by treating *D_i1_*, *D_i2_*, *D_i3_* as the *x*, *y*, *z* Cartesian coordinates.
7. Build a triangle by connecting the three points representing the three subject groups. Rotate and translate the three points, together with point ***Q***, so that the triangle is in the plane *z* = 0. Denote this triangle as ΔEFA_T_.
8. Using the *x*, *y* coordinates of point ***Q*** after transformation, calculate the barycentric coordinates (*λ_ie_*, *λ_if_*, *λ_ia_*) for subject *i* with respect to ΔEFA_T_, using Equations (5) and (6).
9. Convert the barycentric coordinates back to the Cartesian coordinates (*x_i0_*_,_ *y_i0_*) using reference triangle ΔEFA_0_:
  $$x_{i0}=\;\lambda_{ie}x_{e0}+\;\lambda_{if}x_{f0}+\;\lambda_{ia}x_{a0}$$
  $$y_{i0}=\;\lambda_{ie}y_{e0}+\;\lambda_{if}y_{f0}+\;\lambda_{ia}y_{a0}$$
  where (*x_e0_*, *y_e0_*), (*x_f0_*, *y_f0_*), (*x_a0_*, *y_a0_*) are the Cartesian coordinates of the three vertices of ΔEFA_0_.
10. Plot the converted Cartesian coordinates of subject *i*, together with ΔEFA_0_, on the *x-y* plane. The final *x_i0_*_,_ *y_i0_* values are the normalized genetic distances, called GD1 and GD2 scores in this article and in GRAF.
11. The *z* coordinate of point ***Q*** after transformation (step 7), called GD3 score, is also used for plotting results.
12. Calculate the genetic distances *D_i4_* and *D_i5_* from subject *i* to the last two reference populations South Asian and Mexican/Latino. The difference *D_i5_* - *D_i4_*, called GD4 score, is plotted against GD1 scores to separate South Asians from Latin Americans.

A population ID is assigned to each subject based on the ancestor proportions *P_e_*, *P_f_* and *P_a_*, together with GD1 and GD4 scores. The cutoff standards are described in Results.

### Testing GRAF-pop in comparison with existing ancestry-prediction software packages

We compared the performances and prediction accuracies between GRAF-pop and existing software EIGENSTRAT (Price *et al.* 2006), FastPCA (Galinsky *et al.* 2016), SNPweights (Chen *et al.* 2013), and FlashPCA2 (Abraham *et al.* 2017) using the dbGaP studies listed in the first subsection. The first three programs are included in the software package EIGENSOFT 7.1.2 (https://www.hsph.harvard.edu/alkes-price/software/).

We extracted genotypes of the 10,000 fingerprint SNPs and saved them into PLINK sets. We compared the performances of different software packages using the dataset of phs000420.v6.p3, as well as the datasets combined from two or three studies. Missing genotypes were retained in some datasets to evaluate the software packages in the presence of missing genotypes. Since PCA results are displayed in different scales and directions by different PCA programs, PC1 and PC2 values generated by the PCA software packages were normalized using the following method: 1) Genotypes of the HapMap subjects were combined with the datasets to be tested, 2) PC1 and PC2 values were treated as the *x*, *y* coordinates, 3) The centroids of the three HapMap populations CEU, YOR and ASN were calculated and used as the vertices of the reference triangle ΔEFA as mentioned above, 4) The barycentric coordinates with respect to ΔEFA of all subjects were calculated, and converted back to Cartesian coordinates using reference triangle ΔEFA_0_ as mentioned above, and 5) The converted Cartesian coordinates were plotted on scatter plots.

Since GRAF-pop can also estimate the ancestry proportion for each subject, like model-based approaches, we compared GRAF-pop with ADMIXTURE (Alexander *et al.* 2009). Because ADMIXTURE requires that no subjects in the dataset be closely related, we used GRAF (Jin *et al.* 2017) software to find the related subjects and created a dataset including only unrelated subjects to test the software tools.

### Data Availability

Phenotypic and genotypic data required to confirm the results presented in this study are available through the dbGaP Authorized Access System, with the following accessions: phs000050.v1.p1, phs000403.v3.p3, phs000420.v6.p3, phs000456.v1.p1, phs000517.v3.p1, and phs000788.v1.pl. Supplemental material available at FigShare: https://doi.org/10.25387/g3.8061485.

## Results

### Clustering dbGaP subjects and assigning subject populations using GRAF-pop

As of November 15, 2018, dbGaP includes data on 1,987,542 subjects in 1,276 studies and extracted fingerprint genotypes of 1,387,305 subjects from 501 studies; 1,186,387 of these subjects in 478 studies have more than 4,000 fingerprint SNPs genotyped (Table 1). The study-reported populations were submitted to dbGaP as text strings. When non-English characters, UPPER CASE/lower case and singular/plural differences are ignored, there are 264 distinct values for populations.

**Table 1:** Distribution of numbers of fingerprint SNPs with genotypes per subject in dbGaP

#Genotyped SNPs/Subject	#Subjects
101-1000	161860
1001-2000	1209
2001-3000	30441
3001-4000	7408
4001-5000	369599
5001-6000	32640
6001-7000	12242
7001-8000	41849
8001-9000	19207
9001-10000	710850
Total	1387305

**Figure 1:**
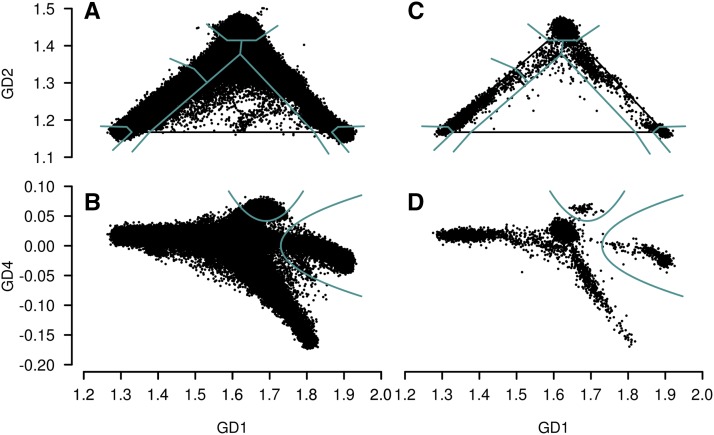
GRAF-pop results of subjects with at least 4,000 fingerprint SNPs genotyped across 478 dbGaP studies. (A, B) All subjects are plotted. (C, D) 10,000 randomly selected subjects are plotted. Cyan lines show the cutoff standards suggested by GRAF-pop to assign dbGaP subjects into GRAF-calculated populations.

We calculated GD1, GD2, GD3, GD4 scores and estimated ancestry proportions for all subjects with fingerprint genotypes extracted. Figure 1 shows the scores of the 1,186,387 subjects with more than 4,000 fingerprint SNPs genotyped.

**Figure 2:**
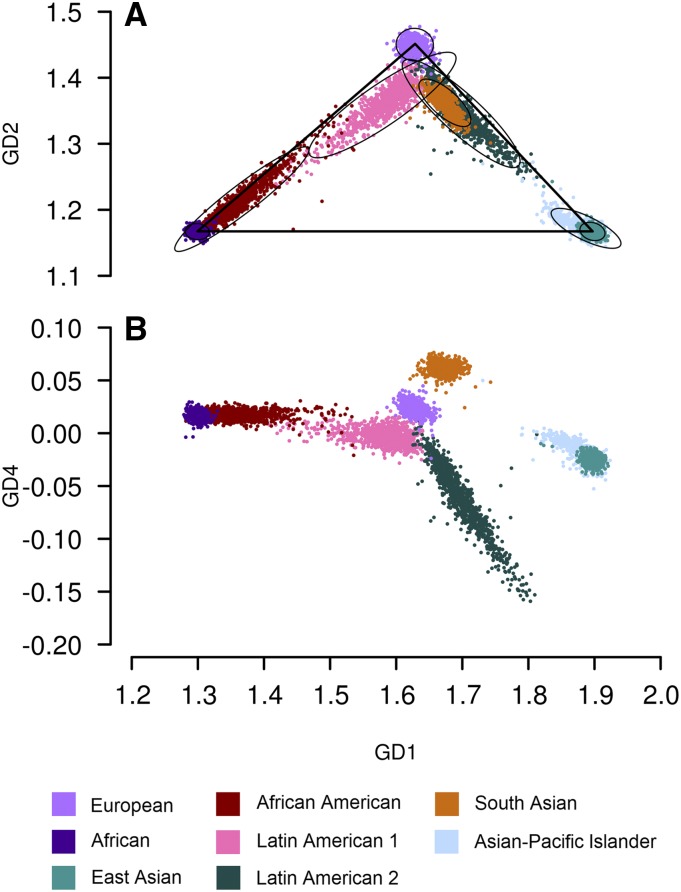
Distribution of GRAF-pop scores of subjects with different groups of study-reported population of subjects across 478 dbGaP studies. Each ellipse in panel (A) shows the area including 95% of the subjects for a certain study-reported population. Only 1,000 randomly selected subjects are plotted for each population to avoid overplotting. Study-reported populations are grouped and harmonized. The population Asian-Pacific Islander does not include South Asian subjects.

Figure 2 shows the distributions of GRAF-pop scores of eight dbGaP study-reported populations: European, African (represented by study-reported populations Ghana and Yoruba), East Asian (represented by Chinese and Japanese), African American (including African), Latin American 1 (represented by Puerto Rican and Dominican), Latin American 2 (represented by Mexican and Mexican American), South Asian (represented by Asian Indian and Pakistani), and Asian-Pacific Islander (including East Asian but not South Asian) . For each population, 1,000 randomly selected subjects are plotted.

In Figure 1, we can see that dbGaP subjects are separated into six clusters by GD1, GD2 and GD4. On the GD2 *vs.* GD1 plots, three main clusters at the vertices of the reference triangle, corresponding to continental populations European, African/African American, and Asian. Below the European cluster, there are two clusters. The left cluster in Figure 1 has more African components, and the location is corresponding to that of the study-reported Latin American 1 in Figure 2. The right cluster is located in the area where the study-reported South Asians, and Latin American 2 are found in Figure 2. On the GD4 *vs.* GD1 plots, the right cluster is separated into three sub-clusters: the top one corresponds to South Asian and the bottom one to Latin American 2. The middle sub-cluster is connected to the Asian-Pacific Islander cluster. The Latin American 1, Latin American 2, and South Asian populations are not homogeneous, so the tightness of the clustering is surprising. We emphasize that the individuals sampled in Figure 2 represent the study-reported populations in dbGaP, but may not comprise a representative sample of these populations as a whole. Naturally, dbGaP staff must check all submissions of data sets, and accept all submissions of data sets that pass quality control, but it would be neither appropriate nor possible (since any research group can submit data) for dbGaP staff to check whether submitted data sets are representative. Analogously, sequence databases maintained at NCBI have a gross excess of sequences from model organisms out of proportion with the diversity of organisms on earth.

We assign each dbGaP subject a unique population ID using the thresholds in Table 2 and Table 3. These simple cutoff lines, plotted in Figure 1 using cyan lines, were set to be able to separate the clusters of dbGaP subjects shown in Figure 1, and the ellipses that include 95% subjects of populations shown in Figure 2. There are no clear separations between African (PopID 2) and African American (PopID 4), and between East Asian (PopID 3) and Asian-Pacific Islanders (not including East Asian, PopID 7). Note that these GRAF-calculated populations, different from the eight study-reported populations used to calculate the ellipse areas in Figure 2, are mutually exclusive. For example, the GRAF-calculated Asian-Pacific Islander population does not include East Asian or South Asian subjects, but in Figure 2, the ellipse area of Asian-Pacific Islander includes subjects from both East Asia and other Asia-Pacific regions.

**Table 2:** Populations assigned by GRAF-pop based on the estimated ancestry proportions

PopID	Population	Cutoff standard
1	European	*P_e_* ≥ 87%
2	African	*P_f_* ≥ 95%
3	East Asian	*P_a_* ≥ 95%
4	African American	40% ≤ *P_f_* < 95% and *P_a_* < 13%
5	Latin American 1	*P_f_* < 40% and *P_e_* < 87% and *P_a_* < 13% and *P_f_* ≥ *P_a_*
6,7,8	(Three populations)	*P_a_* < 95% and *P_e_* < 87% and *P_f_* < 13% and *P_f_* < *P_a_*
9	Other	*P_a_* ≥ 13% and *P_f_* ≥ 13%

**Table 3:** Separating Asians and Hispanics using GD1 and GD4 scores

PopID*	Population	Cutoff standard
7	Asian-Pacific Islander	GD1 > 30 × (GD4)^2^ + 1.73
8	South Asian	GD4 > 5 × (GD1 -1.69)^2^ + 0.042
6	Latin American 2	GD4 < 0 and PopID is not 7

* PopID: ID of population computed by GRAF-pop.

The cutoff lines were determined empirically to aid in grouping the dbGaP subjects. We sought to base the cutoff lines and curves on as few parameters as possible, using the ancestry proportions and GD1-GD4. These lines were selected based on the distribution of dbGaP subjects with known study-reported populations on the GD spaces (Figures 1 and 2). When selecting these cutoff lines, we paid some special attention to the ellipse areas that include 95% of the subjects with the eight known populations. The lines are included in the GRAF software package as a suggested cutoff standard to assign subjects to populations. Other users should only use cutoff lines, as well as the ellipses, as references to compare their own data with dbGaP. GRAF-pop users are supposed to plot the GD scores and ancestry proportions calculated by GRAF-pop and see how the subjects in their own data sets are clustered in the GD space and then select their own standards to group the subjects based on the purposes of data usage.

Table 4 shows the percentages of subjects, among those with ≥ 4,000 fingerprint SNPs genotyped, assigned to different population IDs for different study-reported populations. Table 4 includes the 94 study-reported populations with ≥ 100 subjects with ≥ 4,000 fingerprint SNPs genotyped. We combined the study-reported populations and GRAF-assigned populations into five continental populations and estimated the prediction accuracies of GRAF-pop.

**Table 4:** Prediction accuracies (%) of GRAF-pop for continental populations

Study-reported population	#Subjects	GRAF-pop assigned population						
		European	African	Asian	Latin American 1	Latin American 2	South Asian	Other
**European**	519731	**98.28**	0.12	0.06	0.46	0.69	0.02	0.37
**African**	94366	0.35	**97.46**	0.02	1.49	0.13	0.06	0.50
**Asian**	30946	0.33	0.03	**93.07**	0.06	0.97	**4.62**	0.92
**Hispanic**	39975	8.30	6.21	0.40	**13.26**	**66.94**	0.02	4.88
**South Asian**	4109	0.10	0.10	0.05	0.34	0.00	**98.27**	1.14
**Total**	1186387	74.62	12.10	5.54	1.62	4.45	0.72	0.96

Note: Entries in **bold** are considered correct predictions for the broad classification shown. Here, African means PopID 2 or 4 and Asian means PopID 3 or 7. See Figure S1 for distributions of subjects with different study-reported populations on GRAF-pop scatter plots.

Assuming the study-reported populations correspond to the expected population based on genetic marker data, GRAF-pop correctly determined populations for more than 98% of European and 97% of Asian and African American subjects. The prediction accuracy for South Asian subjects is also greater than 98%, but the number of subjects is small. Approximately 98% of the Asian subjects were predicted by GRAF-pop either as Asians or Asian-Pacific Islanders or South Asians. These predictions were consistent since self-reported Asians can be South Asians. It was most difficult to predict the population of subjects reported as “Hispanics”, or Latin Americans as the harmonized term, which is not surprising considering the complex demographic history of Latin America. Only 80% of study-reported Latin American subjects were predicted by GRAF-pop as Latin American 1 or Latin American 2. Most of the remaining were classified as European, African, or Other, with a small percentage (0.41%) reported by GRAF-pop as Asians or South Asians.

Study-reported populations are sometimes misassigned which makes it difficult to use them to evaluate the performance of GRAF-pop. Figure S1 shows the distributions of subjects with different study-reported populations on GRAF-pop scatter plots. Some subjects with the same study-reported populations are separated by GRAF-pop into several clusters. For example, Figure S1C shows that there is a small cluster in the position near the centroid of Europeans for subjects with a study-reported population of African. It is likely that these subjects are Europeans misassigned as Africans in the dbGaP submission. Figure S1E and S1F shows subjects reported as Asians that are separated into several clusters by GRAF-pop. The top cluster on Figure S1F is in the position near the centroid of South Asian. It is understandable that South Asian participants were reported by the study as Asians. However, the bottom cluster is located where Latin Americans are expected, therefore they are most likely also misassigned. If GRAF-pop predictions are correct, then a possible explanation is that these study-reported populations were estimated by data submitters using genotypes. When PCA is used to infer ancestry and only the first two PC scores are used, it is difficult to distinguish Latin Americans, South Asians and European/East Asian admixtures from one another (see Figure 6B below).

The study-reported populations of the 1000 Genomes Project (Clarke *et al.* 2012) individuals are more reliable. We used genotype data of 2,504 individuals from 1000 Genomes as a test for GRAF-pop. For HapMap defined “super populations” EUR, AFR, EAS, SAS (http://www.internationalgenome.org/category/population/), the accuracies are greater than 99% (Table 5). For the admixed population AMR, more than 81% are predicted as Latin Americans. The super population AFR includes two admixed populations from Americas: ASW (Americans of African ancestry in SW USA) and ACB (African Caribbeans in Barbados). If we exclude these two populations from AFR, then the prediction precision of GRAF-pop in non-admixed continental populations is 100% (see Figures S2A and S2B).

**Table 5:** Prediction accuracies (%) of GRAF-pop using data of 1000 Genomes Project

1000 Genome Population	European	African	Asian	South Asian	Latin American	Other
**EUR**	**100.0**	0.0	0.0	0.0	0.0	0.0
**AFR**	0.0	**99.2**	0.0	0.0	0.2	0.6
**EAS**	0.0	0.0	**100.0**	0.0	0.0	0.0
**SAS**	0.0	0.0	0.0	**99.0**	0.0	1.0
**AMR**	6.1	0.9	0.0	0.0	**81.6**	11.5

Among 1.2 million dbGaP subjects with ≥4000 fingerprint SNPs, about 76% are Europeans, and the percentages of African Americans, Latin Americans and Asians are about 12%, 6% and 5%, respectively.

### Populations of dbGaP subjects predicted by GRAF-pop for each dbGaP study

**Figure 3:**
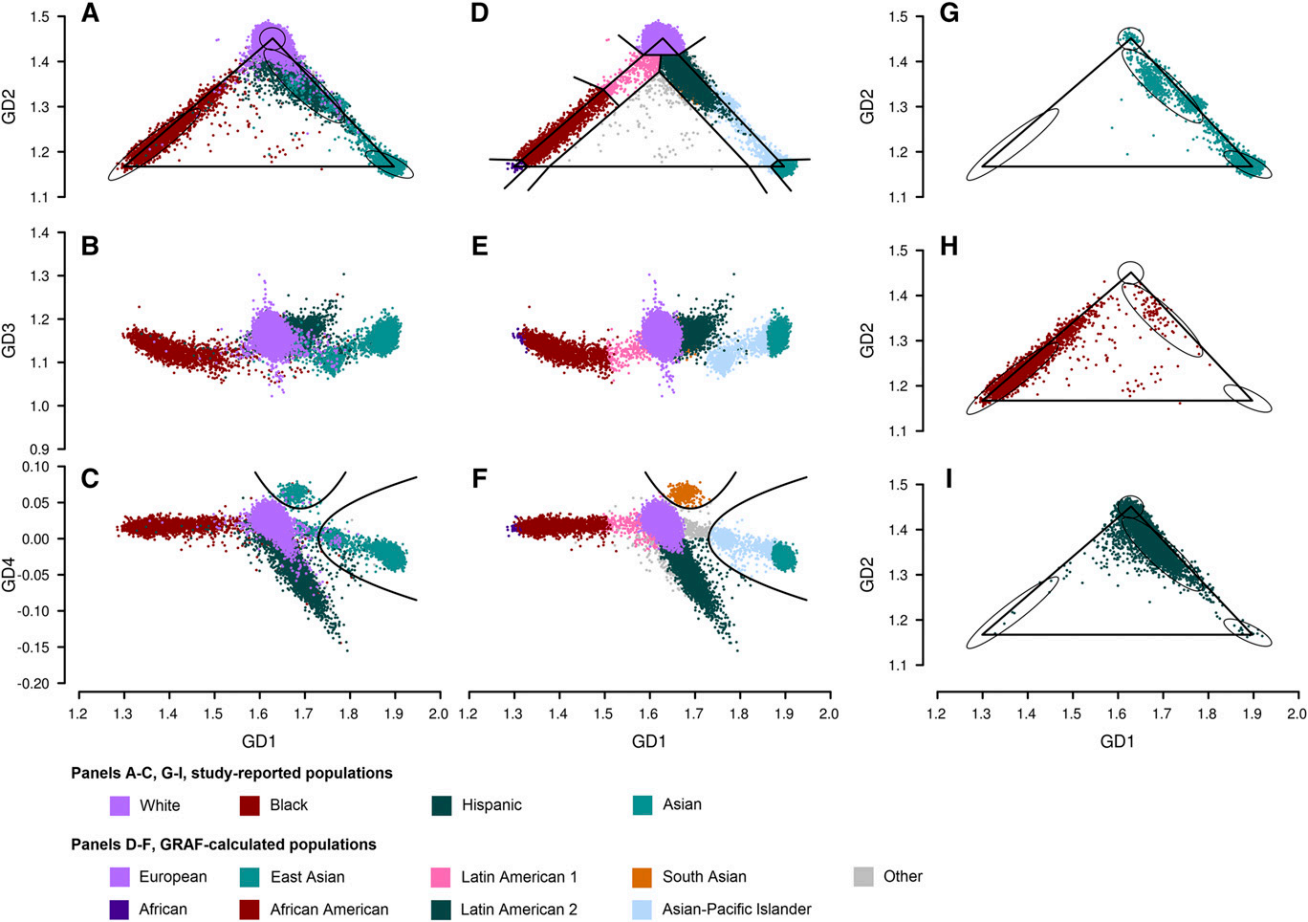
Subject populations determined by GRAF-pop for dbGaP study phs000788.v1: Research Program on Genes, Environment and Health (RPGEH). Each subject has 4028 – 5872 (average 4239) fingerprint SNPs with genotypes. In panels (A-C) and (G-I), study-reported populations are color coded. In panels (D-G), GRAF-calculated populations are color coded. Black cutoff lines are the same as those in Figure 1. Each ellipse shows the area including 95% of the subjects for a study-reported population European, African American, Asian-Pacific Islander, or Latin American 2, same as in Figure 2. Total 78,419 subjects with the following study-reported populations are plotted: White: 65,540 subjects; Asian: 5,510; Hispanic: 4,641; Black: 2,183; Other 545.

For each dbGaP study with genotype data submitted, we use GRAF-pop to estimate subject populations and display the results on the study report page within the dbGaP web site. As an example, Figure 3 shows population reports generated by GRAF-pop for study phs000788.v1.

The study phs000788.v1 is a multi-ethnic study with 78,419 genotyped subjects with an average of 4,259 fingerprint SNPs genotyped per subject. Most subjects are reported as having one of four populations: White, Asian, Hispanic, or Black. Figure 3A shows that most of the subjects are within the areas expected. Each panel G-I plots subjects with one study-reported population. Panel G indicates that some of the study-reported Asians have mostly European backgrounds. The cluster between the European and Asian vertices most likely contains individuals who have both European and Asian ancestry. The subjects within the South Asian expected area are probably South Asians reported as Asians by the data submitter. Figure 3B shows the graph after it is rotated around the bottom of the triangle by 90°; *i.e.*, the GD3 values are shown on the *y*-axis. Hispanics are separated from European/Asian admixtures in the rotated graph. Figure 3C plots GD4 values on the *y*-axis. Study-reported Hispanics are almost completely separated from Asians, including East Asians, European/Asian admixtures, and South Asians. Panels D-F color code the subjects by populations computed by GRAF-pop. The black lines are the cutoff lines to separate different populations. Figure 3F shows that the cluster of study-reported Asians near the expected area for Europeans is composed of South Asians.

**Figure 4:**
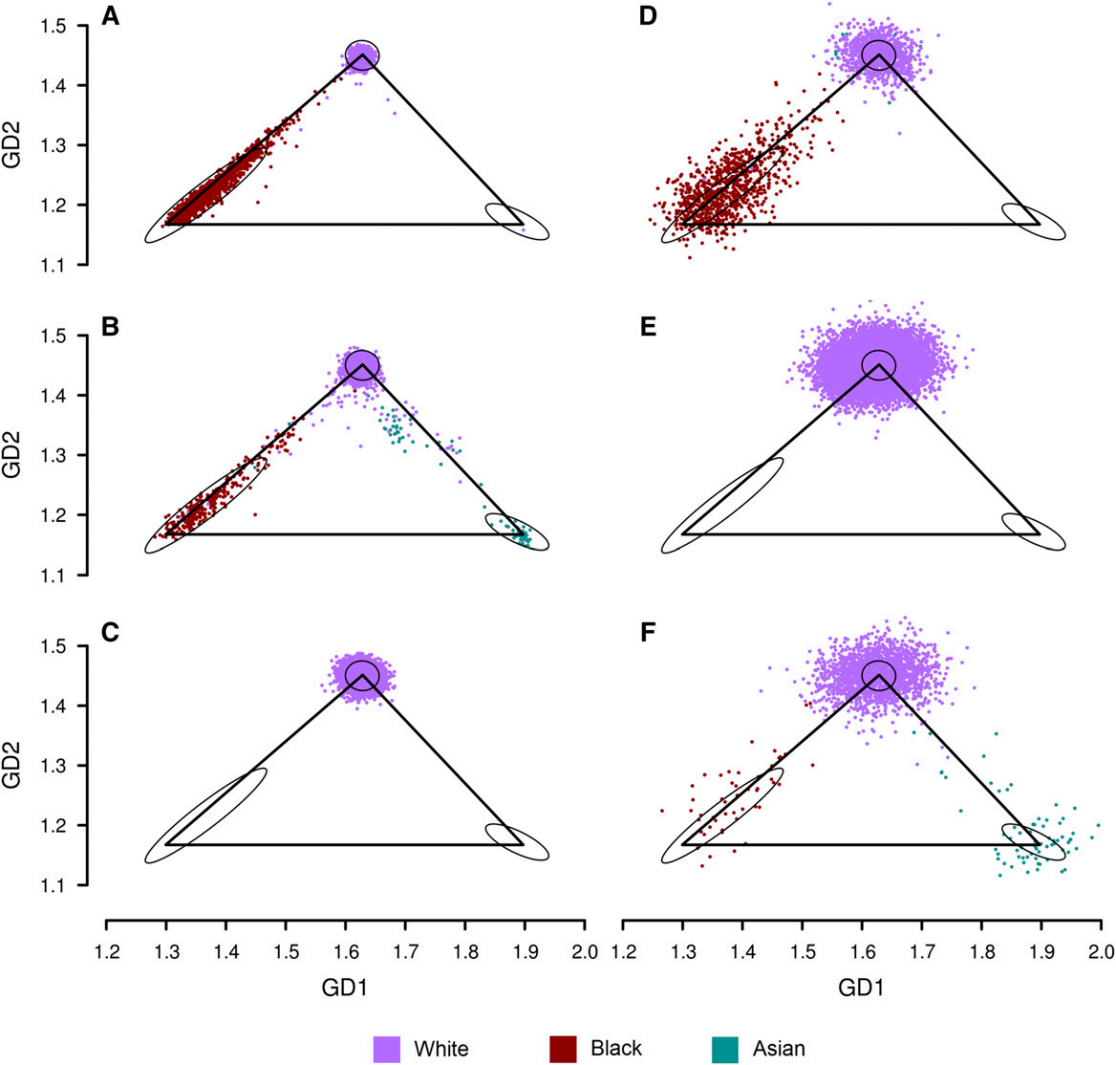
GRAF-pop results of dbGaP studies with different numbers of fingerprint SNPs genotyped. Each panel shows result of one dbGaP study. Study-reported populations are color coded. Each ellipse shows the area including 95% of the subjects for a study-reported population European, African American, Asian-Pacific Islander, same as in Figure 2. (A) Study accession: phs000169.v1. White: 1,663 subjects. Black: 1,139. (B) phs000944.v1. White: 4,132. Black: 2,665. Asian: 81. (C) phs001306.v1. White: 9,695. (D) phs000281.v8. White: 1,020. Black: 884. (E) phs000918.v1. White: 11,402. (F) phs000948.v1. White: 1,786. Black: 49. Asian: 69. From Panels (A) to (F), the average fingerprint SNPs with genotypes per subject are: 9982, 5072, 2040, 471, 244, and 157, respectively.

Different numbers of fingerprint SNPs are genotyped across dbGaP studies. We selected studies with fingerprint SNPs ranging from fewer than 200 to almost all 10,000 SNPs. Figure 4 shows the population reports generated by GRAF-pop and displayed on dbGaP webpages for these studies. European subjects are restricted in a small area if there are more than 2,000 fingerprint SNPs genotyped. The subjects become more widely spread out when there are fewer than 1,000 SNPs genotyped. Roughly 150 fingerprint SNPs genotyped suffice to separate the three main populations (European, African American, and Asian).

### Comparing GRAF-pop to other software tools

We compared performance of GRAF-pop to EIGENSTRAT, FastPCA, and SNPweights included in the EIGENSOFT 7.2.1 package (https://www.hsph.harvard.edu/alkes-price/software/), as well as FlashPCA2 (https://github.com/gabraham/flashpca, version 2.0). We used data from the following five dbGaP studies to test these programs: phs000050.v1.p1, phs000403.v3.p3, phs000420.v6.p3, phs000456.v1.p1 and phs000517.v3.p1. These studies include subjects of all continental populations, and genotype datasets covering both large (>80%) and small (<5%) portions of the fingerprint SNPs. The 270 subjects of phs000050 were from HapMap Phase I and were genotyped using the PERLEGEN-600K platform. We used HapMap data as a cross-dataset positive control because the population assignments are reliable and have been used to test other ancestry inference software. Phs000420 (NHLBI MESA SHARe) is a sub-study of the Multi-Ethnic Study of Atherosclerosis (MESA) Cohort (phs000209.v13.p3) with 8,295 subjects genotyped using the AFFY_6.0 platform. Phs000403 (NHLBI GO-ESP) is another sub-study of MESA, with genotypes obtained using exome sequencing platform Genome Analyzer IIX for 404 subjects. Phs000456 (Risk Assessment of Cerebrovascular Events (RACE) Study) contains 2,493 Pakistani subjects, and phs000517 (Multiethnic Cohort (MEC) Breast Cancer Genetics) includes 3,708 subjects with study-reported populations Black or African American, Latino and Japanese. Both RACE and MEC Breast Cancer studies genotyped subjects using the platform Human660W-Quad_v1_A. Table S2 shows a summary of the five selected studies. For NHLBI MESA SHARe, we combined study-reported populations “African American”, “Black”, and “Black, African-American” into “African American”, and we combined “White, Caucasian” and “European American” into “European American”.

We extracted the genotypes of the 10,000 fingerprint SNPs and filtered out SNPs with genotype missing rates greater than 90%. Two PLINK sets were created to compare the software tools. The first dataset contains combined genotypes from HapMap, NHLBI GO-ESP and NHLBI MESA SHARe. It consists of genotypes of 3,343 SNPs and 8,898 subjects; the counts of subjects from HapMap, NHLBI GO-ESP and NHLBI MESA SHARe are 270, 404 and 8224, respectively. While subjects of HapMap and NHLBI MESA SHARe have genotype missing rates < 0.1%, the NHLBI GO-ESP subjects have genotype missing rate > 95%, which is useful to test the software tools when the genotype missing rates are high. The second dataset was generated by combining genotypes of HapMap, RACE Study and MEC Breast Cancer, containing genotypes of 7,970 SNPs and 6,616 subjects, with genotype missing rate < 5% for all subjects.

We deliberately chose to show the performance of GRAF-pop on real individual data sets in dbGaP, rather than on blended or simulated data sets. The decision to use individual data sets refleects the reality of the quality-control process. Each data set is idiosyncratic and each submitting group may use different procedures to assign study-reported populations and may make unique errors in managing data. Some of the systematic submitter errors that GRAF-pop has detected would have been quite unrealistic to simulate.

**Figure 5:**
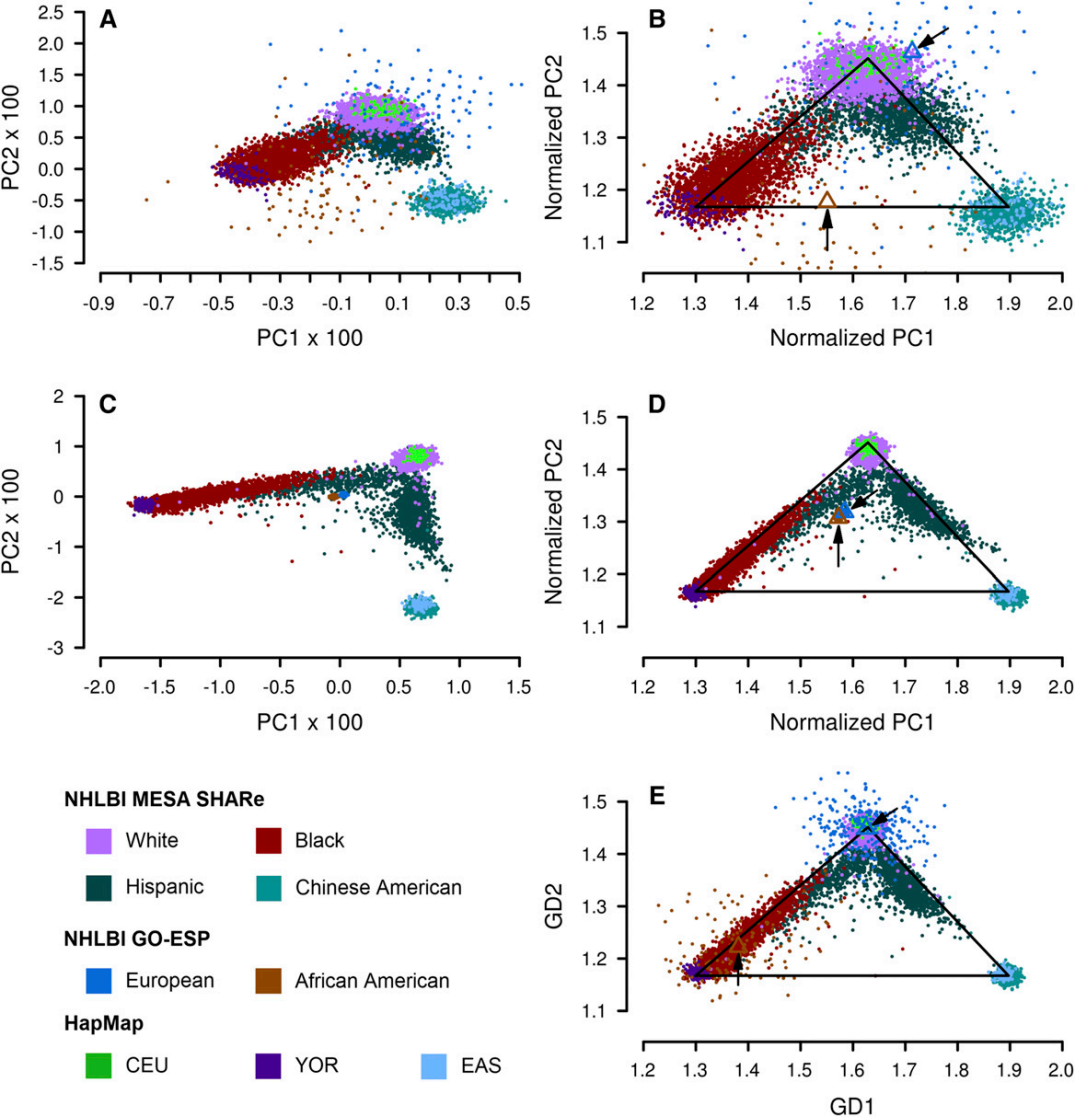
Results generated by applying SNPweights, EIGENSTRAT and GRAF-pop on the combined dataset of phs000050.v1 (HapMap), phs000403.v3 (NHLBI GO-ESP) and phs000420.v6 (NHLBI MESA SHARe) to infer subject ancestry. Average number of SNPs with genotypes per subject in the test data set: HapMap and NHLBI MESA SHARe: 3,433; NHLBI GO-ESP: 160. For ease of comparison, SNPweights and EIGENSTRAT results are plotted on the three-population triangle as explained in Methods. (A) SNPweights, PC2 *vs.* PC1. (B) SNPweights, after normalization onto the GRAF-pop results. (C) EIGENSTRAT, PC2 *vs.* PC1. (D) EIGENSTRAT, after normalization. (E) GRAF-pop, GD2 *vs.* GD1. The two small triangles on Panels (B, D, E), each of which is pointed by a black arrow, show the centroids of the subject locations of the two study-reported populations in NHLBI GO-ESP predicted by each software tool: blue triangle for European, brown for African American. Since the subjects in NHLBI GO-ESP have very high genotype missing rates, these centroids indicate whether a software tool can estimate subject ancestry unbiasedly in the presence of missing genotypes.

Figure 5 shows results from SNPweights, EIGENSTRAT, and GRAF-pop using the first dataset. Comparing the normalized results of EIGENSTRAT (Figure 5D) and those of GRAF-pop (Figure 5E), we can see that both methods can separate the continental populations from one another well, when genotype missing rates are low. The resolutions of EIGENSTRAT and GRAF-pop are similar, and both are much higher than the resolution of SNPweights (Figure 5A-B).

When genotype missing rates are high, as in NHLBI GO-ESP, EIGENSTRAT did not determine the populations correctly. All subjects from NHLBI GO-ESP, both European Americans or African Americans, were placed near the center (Figure 5C-D). Similar results were obtained by running EIGENSTRAT with option *lsqproject*. SNPweights put these subjects in widely spread areas, with centroids of European Americans and African Americans far from the centroids of the same populations in other studies (Figure 5A-B). Only GRAF-pop could clearly separate African Americans from European Americans, and the centroids of these two populations were close to those from other studies (see the two small triangles pointed by black arrows in panels B, D, E).

**Figure 6:**
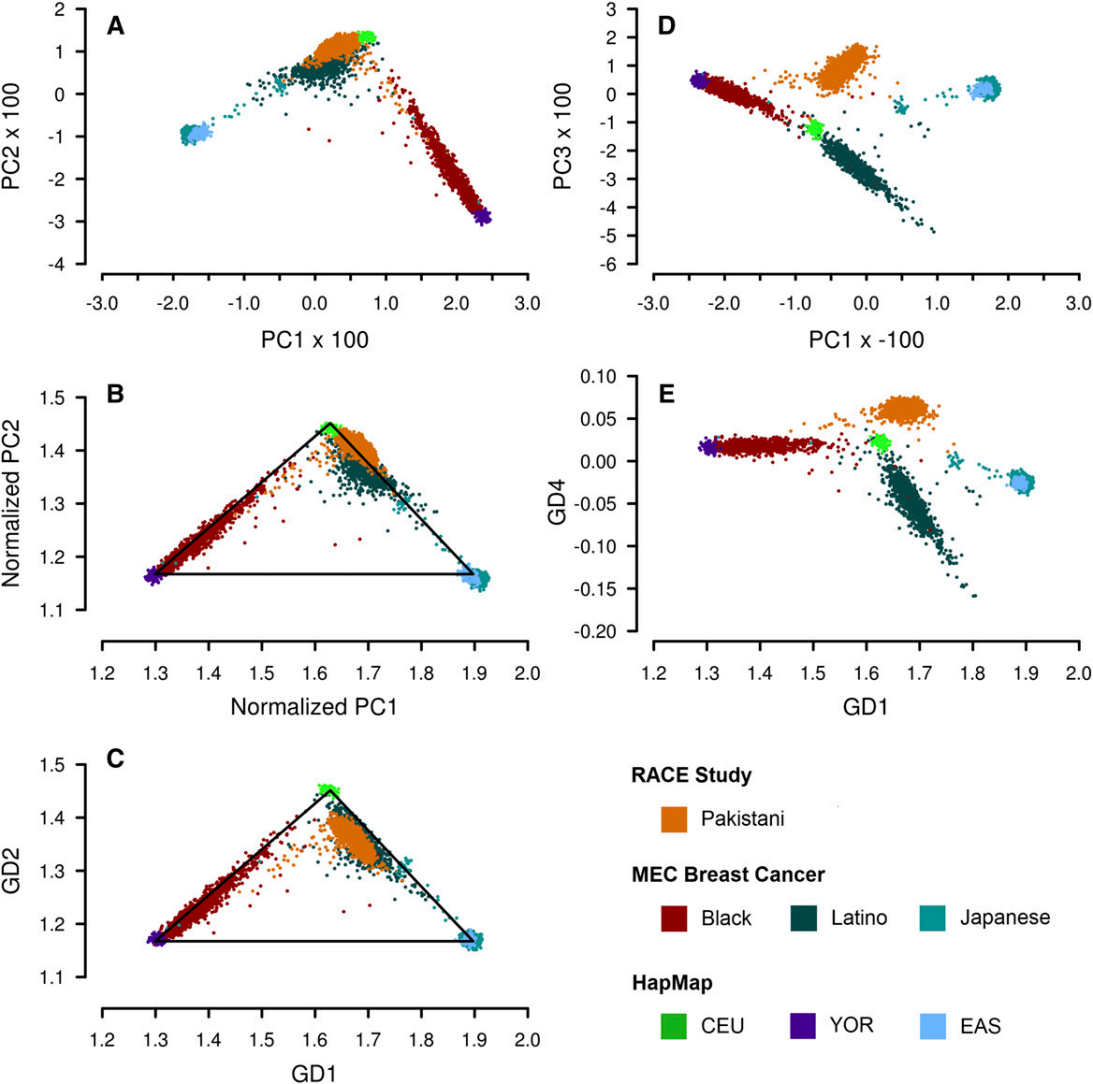
Results generated by applying EIGENSTRAT and GRAF-pop on the combined dataset of phs000050.v1 (HapMap), phs000456.v1 (Risk Assessment of Cerebrovascular Events (RACE) Study) and phs000517.v3 (Multiethnic Cohort (MEC) Breast Cancer Genetics) to infer subject ancestry. 7,790 SNPs are included in the test data set, with genotype missing rate less than 5% for each subject. (A) EIGENSTRAT, PC2 *vs.* PC1. (B) EIGENSTRAT, PC2 *vs.* PC1, after normalization. (C) GRAF-pop, GD2 *vs.* GD1. (D) EIGENSTRAT, PC3 *vs.* PC1. (E) GRAF-pop, GD4 *vs.* GD1.

Figure 6 compares the results of EIGENSTRAT and GRAF-pop on the combined dataset of HapMap, RACE Study and MEC Breast Cancer. Panels A-C show that study-reported Europeans, African Americans, Asians, and Hispanics can be well separated from one another using PC1 and PC2, or the GD1 and GD2 scores of GRAF-pop. The resolutions of these two programs were close. However, South Asians (Pakistanis in this test) could not be well separated from Hispanics. Figure 6D shows that South Asians could be separated from Hispanics using PC3 of EIGENSTRAT. Similarly, Figure 6E shows that these two populations could be distinguished from each other using the GD4 scores of GRAF-pop.

We also tested FastPCA and FlashPCA2. For all the datasets tested, FastPCA and FlashPCA2 generated results almost identical to EIGENSTRAT, which is not surprising since they all use PCA.

We compared the running times of these software tools on an Intel Xeon machine with 16 2.67 GHz CPUs using the combined dataset of HapMap, NHLBI GO-ESP and NHLBI MESA SHARe (8,898 individuals). The running times were: FlashPCA2, 8 sec; FastPCA, 22 sec; GRAF-pop, 75 sec; SNPweights, 9 min; EIGENSTRAT, 94 min. Only 3,343 SNPs with low genotype missing rates are included in the dataset. FlashPCA2 and FastPCA only need to analyze genotypes of these SNPs, but GRAF-pop checks genotypes of all 10,000 fingerprint SNPs.

**Figure 7:**
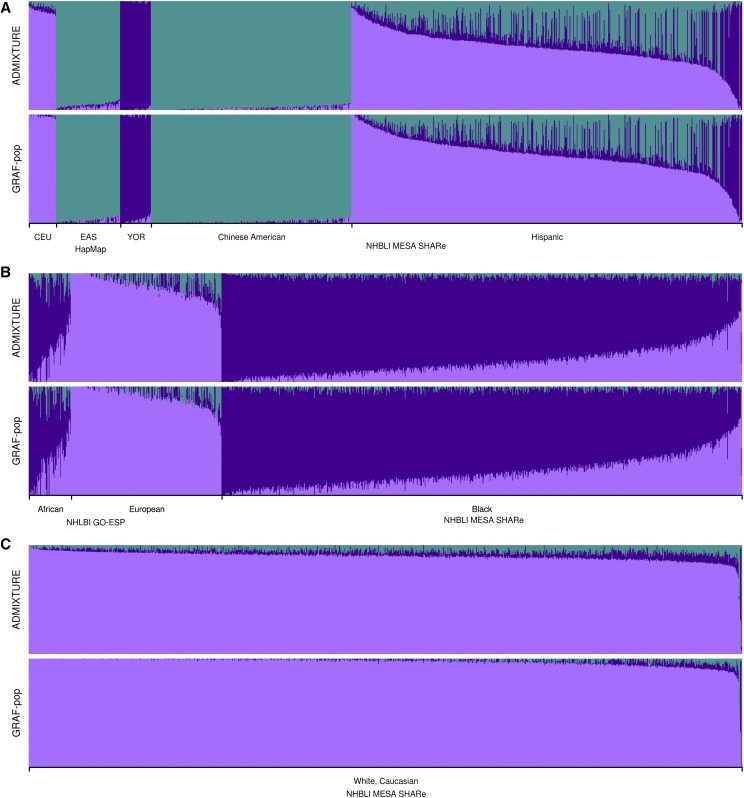
Comparison of population structure determined using ADMIXTURE and GRAF-pop. The data set tested contains genotypes of 3,433 well separated SNPs and 6,794 unrelated subjects from dbGaP studies HapMap, NHLBI MESA SHARe and NHLBI GO-ESP. Subjects from NHLBI GO-ESP have average 160 SNPs with genotypes, while subjects from the other two studies have average genotype missing rate < 0.1%. Each bar represents one subject, and results of same subject are plotted on the same horizontal position to compare the two software tools. For NHLBI MESA SHARe, only about 40% randomly selected subjects are plotted. Three colors are used to represent the three ancestry components: light purple: European; purple: African; mid blue: East Asian.

We also compared GRAF-pop with the widely-used, model-based method ADMIXTURE (v.1.3.0, http://software.genetics.ucla.edu/admixture/download.html). The above first dataset containing combined genotypes from HapMap, NHLBI GO-ESP and NHLBI MESA SHARe was used for testing ADMIXTURE. Since many of the 14,003 subjects in the dataset are closely related, the data set had to be reduced to meet ADMIXTURE’s requirements. We used our GRAF software to create a subset of data of 6,974 subjects, among which no two subjects are closely related (*i.e.*, with HGMR < 15%). ADMIXTURE was run using option *k* = 3. It converged in 36 iterations with running time 139 sec. GRAF-pop spent 36 sec to calculate the results. Figure 7 shows that the results obtained using ADMIXTURE and GRAF-pop are very similar, including the NHLBI GO-ESP subject with very high genotype missing rates. Both methods can easily cluster the non-admixed populations correctly. Out of the 1,410 subjects with study-reported population Hispanic, 74 and 6 subjects are determined to be Europeans and Asians, respectively, by GRAF-pop using the default cutoff values. For those subjects, very high European (86%) and Asian (87%) ancestry proportions are also predicted by ADMIXTURE.

### Applying GRAF-pop in dbGaP curation

During development, GRAF-pop was used informally to harmonize population values for searching and as a quality control tool to check study-reported populations in newly submitted dbGaP studies. Advice from curators aided improvements to the visualization tools illustrated in the Figures. Since February 2018, GRAF-pop has been used formally to check the genotype data and population assignments before each new study is publicly released by dbGaP.

Populations predicted by GRAF-pop and those reported in the submitted files are displayed and compared on a webpage for curators and submitters to use to find potential errors in the phenotype and genotype files. The page also highlights missing information to be filled in before the submission is complete.

Below is an example URL for one of these webpages:

https://www.ncbi.nlm.nih.gov/projects/gap/population/cgi-bin/StudySubjectAncestry.cgi?phs=420&version=6&exp1=1&exp2=1&exp3=1

Our expectation has been that GRAF-pop would catch omissions and errors in submitters’ assignment of subject populations. GRAF-pop has already also identified some inconsistencies in the content of PLINK-formatted genotype files. GRAF-pop can flag files that have a syntactically valid PLINK format, but have incorrect marker assignments, which leads to unexpected GRAF-pop predictions of subject population.

## Discussion

PCA is a linear algebraic method applied in many scientific disciplines (Jolliffe and Cadima 2016), and has long been used to analyze genetic data (Menozzi *et al.* 1978). Since the development of EIGENSOFT (Patterson *et al.* 2006; Price *et al.* 2006), the use of PCA has become central to many ancestry inference methods. However, PCA has two important limitations when applied to ancestry inference.

One limitation is that PCA is a dimensionality reduction method without a statistical model. Mcvean (2009) has shown that PCA scores give an accurate representation of the parameters of the coalescent process. However, PCA scores do not have physical meaning. They can only be used to separate individuals within the dataset being analyzed (Lee *et al.* 2010), and therefore, cannot be generalized across datasets. Recent methods, such as SNPweights (Chen *et al.* 2013) and FastPop (Li *et al.* 2016) mitigate this limitation by using reference panels as well as algorithmic steps before and after PCA.

The second limitation is that PCA has difficulty with missing data (Stanimirova *et al.* 2007). Most of the existing PCA ancestry inference approaches filter out individuals and SNPs with missing rates greater than some threshold, usually 5% or 1%. Missing values remaining in the datasets are then replaced with the mean value (Price *et al.* 2006; Chen *et al.* 2013; Li *et al.* 2016; Byun *et al.* 2017), which shifts individuals toward the centroid in the scatter plot generated by PCA. Li *et al.* noticed that false populations were reported by FastPop when samples with genotype missing rates higher than 5% were included (Li *et al.* 2016).

To infer the population assignment for dbGaP subjects genotyped on various platforms, we have developed an inference method named GRAF-pop to improve quality control in curation, and harmonize subject population values across studies. It uses a distance-based approach that overcomes these two limitations of PCA. Instead of comparing an individual’s genotypes with those of other individuals in the dataset being analyzed, GRAF-pop compares the individuals to fixed sets of reference panels with known populations. Since the number of human populations at a coarse level is limited, the genetic distance data generated by GRAF-pop is low-dimensional, and PCA is not necessary. GRAF-pop utilizes dbGaP subjects with known populations as references. Due to the paucity of Native American samples, we calculate the genetic distances to three reference continental populations: European, African, and Asian, and obtain results of data in 3D. By projecting the initial prediction onto the fixed EFA triangle, GRAF-pop produces results that can be generalized across different datasets. Figure 5 and Figure 6 show that the GRAF-pop GD2 *vs.* GD1 plots, which show the EFA projection, separate continental populations from one another almost as well as the PC2 *vs.* PC1 plots, which show the PCA 2D projection to the plane of the first two PCs. Using the EFA triangle, we can also estimate the ancestral proportions by calculating barycentric coordinates.

Barycentric coordinates were developed by August Ferdinand Möbius (1790-1868) in his book “*Der barycentrische calcul*” (1827) (Ungar 2010). Nowadays, Möbius is better known for discovering the non-orientable three-dimensional shape known as the Möbius strip, which has been cut in three pieces and stylized into the universal symbol for recycling. In that spirit, our work recycles the technique of barycentric coordinates for application to ancestry inference. Readers may have seen barycentric coordinates used in computer graphics and animation to interpolate colors over any convex shape and to deform shapes smoothly, and for other applications (Warren *et al.* 2007; Skala 2008; Weber *et al.* 2009).

For a triangle on a 2D plane, barycentric coordinates solve the following problem: given a point, *p*, inside the triangle, find the unique masses of three objects summing to 1 such that when they are placed on the three vertices, *p* is the center of mass. Applying barycentric coordinates to the EFA triangle and treating the mass proportions as the ancestral proportions, we can estimate the population components for each individual based on the projection of that individual’s distance data relative to the vertices of the reference triangle.

Both SNPweights (Chen *et al.* 2013) and FastPop (Li *et al.* 2016) estimate subject ancestral proportions using distances from the subject to the centroids of reference populations. Surprisingly, these two methods give two *different algebraic* solutions. In each paper, the necessary algebra is shown, but without any justification or comparison to the alternative. One of our contributions is to point out that the method of SNPweights has a natural *geometric* interpretation as barycentric coordinates. Usage of barycentric coordinates allows GRAF-pop to characterize other populations beyond E, F, A, geometrically (see Results), unlike SNPweights and FastPop.

GRAF-pop also uses barycentric coordinates to treat the missing data problem. By using reference panels, GRAF-pop can infer ancestry proportions for individuals independent of others in the dataset. In contrast to SNPweights and FastPop, which require that the same SNPs be genotyped in all target individuals, GRAF-pop does not need to impute missing data. GRAF-pop simply skips the SNPs without genotypes. When the average genetic distances from individuals in each vertex subpopulation to the reference population are available for the subset of genotyped SNPs, then barycentric coordinates can be calculated using these mean distances as the reference triangle. *Expected* mean values for a subject group can be predicted using the allele frequencies of the group, for any combination of SNPs. We use the expected mean genetic distances from the three subject groups to the three reference populations to build the reference triangle. The GD2 *vs.* GD1 scatter plots of ancestry results of dbGaP studies calculated using GRAF-pop (*e.g.*, Figure 4 and Figure 5) confirm that the centroid of each population remains nearly unaffected when different numbers of SNPs are missing.

GD1 and GD2 separate continental populations (European, African/African American, Asian-Pacific Islander) from one another (Figures 2A). Latin Americans comprise an admixed population with genetic contributions from European, African and/or Native American populations. Most subjects self-described as “Hispanics” or “Latinos” in the United States either have large proportions of European and Native American components, or European and African components (Bryc *et al.* 2010; Bryc *et al.* 2015). Ancestry inference using GRAF-pop shows similar results. On the GD2 *vs.* GD1 plots, study-reported Hispanics and Latinos form two sweeping clusters (Figures 2, 3): one with more African proportion (labeled Latin American1 by GRAF-pop) than the other (labeled Latin American2).

The following three admixed populations are more difficult to distinguish: Latin American 2, South Asian, and individuals experiencing recent European/East Asian admixture. On the GD2 *vs.* GD1 graph, they show up roughly in the same region of the graph. These populations can be separated by GD4, which is the difference of the genetic distances from each individual to the reference populations of Latin American 2 and South Asian (Figures 1-2). Although not normalized, GD4 is not sensitive to non-random missing genotypes. Indeed, any such difference of two genetic distances with two reference populations can be rewritten as:

$$D_{ij}-D_{ik}=-\frac{1}{S}\;\overset{S}{\underset{l=1}{\sum }}\left[g_{il}\;\ln\left(\frac{p_{jl}}{p_{kl}}\right)+(2-g_{il})\ln\left(\frac{q_{jl}}{q_{kl}}\right)\right]\;$$ (7)

Note that the last term in Equation (2) cancels in the subtraction since the subject genotype *g_il_* is the same no matter what reference population is used. Only the *ratios* of the allele frequencies are used for calculating the difference, and hence the genetic distance differences are robust against missing data.

GRAF-pop has been used by dbGaP curators and submitters as a quality assurance tool, and to generate displays of the ancestry inference results to potential requestors of the data. For example, on the study report page of phs000001.v1.p1: https://www.ncbi.nlm.nih.gov/projects/gap/cgi-bin/study.cgi?study_id=phs000001.v1.p1, there is an “Ancestry Component” link that opens the webpage showing GRAF-pop results:

https://www.ncbi.nlm.nih.gov/projects/gap/population/cgi-bin/StudySubjectAncestry.cgi?phs=1&version=1&exp1=1&exp2=1&exp3=1. These displays can call attention to unusual results, *e.g.*, abnormal data distributions that manifest as discordance between study-reported populations and those reported by GRAF-pop.

Two limitations of the current implementation of GRAF-pop are: 1) it relies on the 10,000 dbGaP fingerprint SNPs, which were selected for other purposes (Jin *et al.* 2017) and 2) only five reference populations are used to calculate the genetic distances, and these do not include Native American. After applying GRAF-pop to hundreds of dbGaP studies, we noticed that about 2,000 fingerprint SNPs are needed to obtain good resolution in separating continental populations. Figure 4 shows some examples. When fewer than 1,000 fingerprint SNPs have genotypes, PCA tools such as EIGENSTRAT will perform better than the current version of GRAF-pop, since they can use all available SNPs in the data sets, although some SNPs within linkage disequilibrium (LD) regions might need to be pruned. Missing data can be imputed if possible. However, GRAF-pop introduces a new method of inferring ancestry that does not use PCA. In the future, we would like to improve the software using more SNPs. Theoretically, GRAF-pop can be implemented using all the variants in the whole genome, so that all SNPs available in any given data set can be used to do ancestry inference. Similar LD-pruning can be done for GRAF-pop as for PCA. However, imputation would not be needed no matter how many genotypes are missing in the data sets. Therefore, we did not and will not try to do imputation for the current and future versions of the GRAF-pop software.

We also would like to improve GRAF-pop by using more reference populations, especially Native American. Some researchers tried to estimate allele frequencies of Native Americans (Gravel *et al.* 2013). When these allele frequencies become available, we can use the centroid of Native Americans as a fourth vertex to build a tetrahedron instead of the EFA triangle, and continue to use barycentric coordinates to estimate ancestry proportions.

Most existing ancestry inference methods analyze genotypes as “unsupervised inference”. Some investigators have developed methods that instead include “supervised analyses”, making use of genotype data of individuals with known ancestral histories (Alexander and Lange 2011). Some methods, including GRAF-pop, do not require that the raw genotypes in the reference panels be publicly available. Instead, only the allele frequencies or pre-computed SNP weights of the reference panels are needed to infer ancestry (Chen *et al.* 2013; Bansal and Libiger 2015; Li *et al.* 2016), which increases efficiency. As stated by Bansal and Libiger, the accuracies of these methods depend on the availability of accurate allele frequencies for reference populations. Large public databases and genotyping consortia will participate in creating future reference panels that will enable improvements in GRAF-pop.
